# An Entomopathogenic Strain of *Beauveria bassiana* against *Frankliniella occidentalis* with no Detrimental Effect on the Predatory Mite *Neoseiulus barkeri*: Evidence from Laboratory Bioassay and Scanning Electron Microscopic Observation

**DOI:** 10.1371/journal.pone.0084732

**Published:** 2014-01-14

**Authors:** Shengyong Wu, Yulin Gao, Yaping Zhang, Endong Wang, Xuenong Xu, Zhongren Lei

**Affiliations:** 1 State Key Laboratory for Biology of Plant Diseases and Insect Pests, Institute of Plant Protection, Chinese Academy of Agricultural Sciences, Beijing, P.R. China; Ghent University, Belgium

## Abstract

Among 28 isolates of *Beauveria bassiana* tested for virulence against *F. occidentalis* in laboratory bioassays, we found strain SZ-26 as the most potent, causing 96% mortality in adults at 1×10^7^ mL^−1^conidia after 4 days. The effect of the strain SZ-26 on survival, longevity and fecundity of the predatory mite *Neoseiulus* (*Amblyseius*) *barkeri* Hughes were studied under laboratory conditions. The bioassay results showed that the corrected mortalities were less than 4 and 8% at 10 days following inoculation of the adult and the larvae of the predator, respectively, with 1×10^7^ conidia mL^−1^ of SZ-26. Furthermore, no fungal hyphae were found in dead predators. The oviposition and postoviposition durations, longevity, and fecundity displayed no significant differences after inoculation with SZ-26 using first-instar larvae of *F. occidentalis* as prey in comparison with untreated predator. In contrast, the preoviposition durations were significantly longer. Observations with a scanning electron microscope, revealed that many conidia were attached to the cuticles of *F. occidentalis* at 2 h after treatment with germ tubes oriented toward cuticle at 24 h, penetration of the insect cuticle at 36 h, and finally, fungal colonization of the whole insect body at 60 h. In contrast, we never observed penetration of the predator's cuticle and conidia were shed gradually from the body, further demonstrating that *B. bassiana* strain SZ-26 show high toxicity against *F. occidentalis* but no pathogenicity to predatory mite.

## Introduction

Western flower thrips, *Frankliniella occidentalis* (Pergande) (Thysanoptera: Thripidae), is regarded as an important economic pest of a wide range of agricultural and horticultural crops worldwide [1]–[4]. Because *F. occidentalis* has developed a high level of resistance to many chemical pesticides [5]–[7], it is essential to adopt a biological control program for this pest. The predatory mite *Neoseiulus* (*Amblyseius*) *barkeri* (Hughes) (Acarina: Phytoseiidae) and the entomopathogenic fungus *Beauveria bassiana* (Balsamo) Vuillemin have been shown to be potential biological control agents of *F. occidentalis* [7], [8]–[11].

*N. barkeri* has been successfully employed for reducing populations of *F. occidentalis* in crops, such as strawberries and cucumbers [12], [13]. However, the control efficiency for thrips is limited because the mite prefers to prey only on larval stages of thrips [14], [15]. The application of the entomopathogenic fungus *B.bassiana* against *F. occidentalis* results in high rates of mortality in laboratory screenings and greenhouse conditions [16]–[18]. In order to obtain the highest efficiency in controlling *F. occidentalis*, it is suggested that *B.bassiana* should be applied along with the releases of predatory mites under field conditions [9], [18]. Therefore, evaluating the compatibility of applying *B. bassiana* and predators to control *F. occidentalis* is a critical issue for the implementation of IPM programs. A better understanding of the factors that minimize undesirable effects of insect pathogens on natural enemies could improve their integrated utilization against pest insects [19].

Most previous research has been designed to evaluate the effects of pathogens on predators directly by exposing predators to pathogen residues or by topical application, and then studying factors such as predator mortality and behavior, or indirectly by allowing predation on fungal-infected preys, or assessing predator-prey abundance in experimental crops. [9], [20]–[22]. Recent studies have focused on effects on fecundity of predators [23], [24]. We determined the compatible utilisation of *B. bassiana* strain SZ-26 and *N. barkeri* by studying the effect on the longevity and fecundity of predatory mites when offered first-instar thrips as prey. Furthermore, there are no reports for the micromorphological observations of fungal conidial inoculation processes on this predator. Thus, we studied the mortality of larval and adult *N. barkeri* and *F. occidentalis* exposed to *B. bassiana* strain SZ-26 and compared the fungal infection process in the predator and thrips by scanning electron microscopy (SEM). This information will enhance our understanding of the interactions between two biological control agents and help to determine the possibility of concomitant application of both in *F. occidentalis* classical biocontrol programmes.

## Results

### Screening fungal isolates

**Figure 1:**
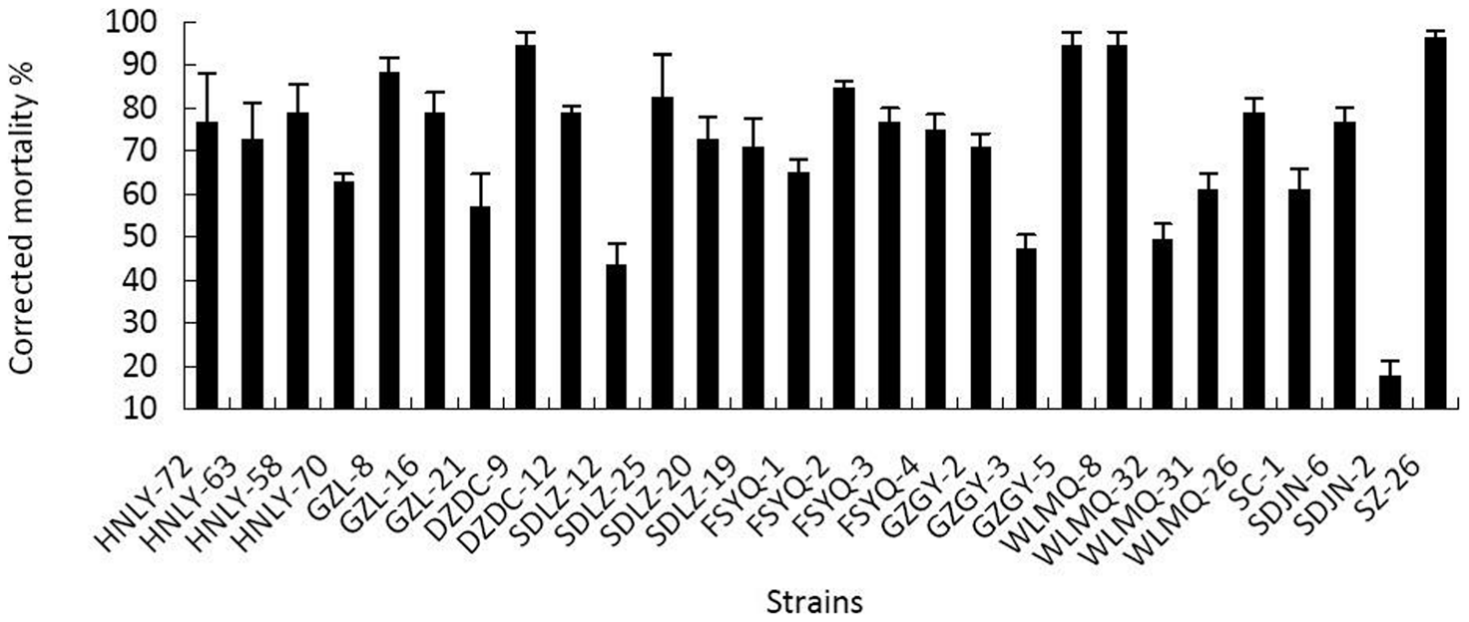
Corrected mortality of 28 isolates against adult *T. tabaci* 4 days post treatment in the laboratory. Data are expressed as means ± SEM based on 3 replications, each consisting of 20 adults. All strains were tested at 1×10^7^ conidia mL^−1^.

Of the 28 strains of *B. bassiana* (Table 1) tested at 1×10^7^ conidia mL^−1^ in the laboratory, the SDLZ-12 strain caused only 43% mortality after 4 days, while strain SZ-26 killed the highest percentages with 96% mortality (F = 11.212, p<0.001) (Fig. 1). Strain SZ-26 was identified as the most virulent strain and was selected for further evaluation on the predatory mite, *N. barkeri*.

**Table 1:** Origin of *Beauveria bassiana* isolates screened against the western flower thrips, *Frankliniella occidentalis.*

Fungal isolates	Host or source of origin	Site origin (Collected date)
SZ-26	*Ostrinia nubilalis* (Lepidoptera: Pyralidae)	Suizhong, Liaoning (2011)
HNLY-58	*Ostrinia furnacalis* (Lepidoptera: Pyralidae)	Luoyang, Henan (2010)
HNLY-63	*Ostrinia furnacalis* (Lepidoptera: Pyralidae)	Luoyang, Henan (2010)
HNLY-70	*Ostrinia furnacalis* (Lepidoptera: Pyralidae)	Luoyang, Henan (2010)
HNLY-72	*Ostrinia furnacalis* (Lepidoptera: Pyralidae)	Luoyang, Henan (2010)
GZL-8	*Ostrinia furnacalis* (Lepidoptera: Pyralidae)	Gong Zhuling, Jilin (2011)
GZL-16	*Ostrinia furnacalis* (Lepidoptera: Pyralidae)	Gong Zhuling, Jilin (2011)
GZL-21	*Ostrinia furnacalis* (Lepidoptera: Pyralidae)	Gong Zhuling, Jilin (2011)
FSYQ-1	*Trialeurodes vaporariorum* (Westwood)	Yongqing, Hebei(2011)
FSYQ-2	*Trialeurodes vaporariorum* (Westwood)	Yongqing, Hebei(2011)
FSYQ-3	*Trialeurodes vaporariorum* (Westwood)	Yongqing, Hebei(2011)
FSYQ-4	*Trialeurodes vaporariorum* (Westwood)	Yongqing, Hebei(2011)
SCWJ-1	*Ostrinia nubilalis* (Lepidoptera: Pyralidae)	Wenjiang, Sichuang (2011)
SDJN-2	*Ostrinia nubilalis* (Lepidoptera: Pyralidae)	Jinan, Shandong (2011)
SDJN-6	*Ostrinia nubilalis* (Lepidoptera: Pyralidae)	Jinan, Shandong (2011)
SDLZ-12	*Ostrinia furnacalis* (Lepidoptera: Pyralidae)	Laizhou, Shandong (2010)
SDLZ-19	*Ostrinia furnacalis* (Lepidoptera: Pyralidae)	Laizhou, Shandong (2010)
SDLZ-20	*Ostrinia furnacalis* (Lepidoptera: Pyralidae)	Laizhou, Shandong (2010)
SDLZ-25	*Ostrinia furnacalis* (Lepidoptera: Pyralidae)	Laizhou, Shandong (2010)
GZGY-2	*Ostrinia furnacalis* (Lepidoptera: Pyralidae)	Guiyang Guizhou, (2010)
GZGY-3	*Ostrinia furnacalis* (Lepidoptera: Pyralidae)	Guiyang, Guizhou (2010)
GZGY-5	*Ostrinia furnacalis* (Lepidoptera: Pyralidae)	Guiyang, Guizhou (2010)
DZDC-9	*Ostrinia furnacalis* (Lepidoptera: Pyralidae)	Decheng, Dezhou(2012)
DZDC-12	*Ostrinia furnacalis* (Lepidoptera: Pyralidae)	Decheng, Dezhou(2012)
WLMQ-8	*Ostrinia furnacalis* (Lepidoptera: Pyralidae)	Urumqi, Xinjiang (2012)
WLMQ-31	*Ostrinia furnacalis* (Lepidoptera: Pyralidae)	Urumqi, Xinjiang (2012)
WLMQ-32	*Ostrinia furnacalis* (Lepidoptera: Pyralidae)	Urumqi, Xinjiang (2012)
WLMQ-26	*Ostrinia furnacalis* (Lepidoptera: Pyralidae)	Urumqi, Xinjiang (2012)

**Figure 2:**
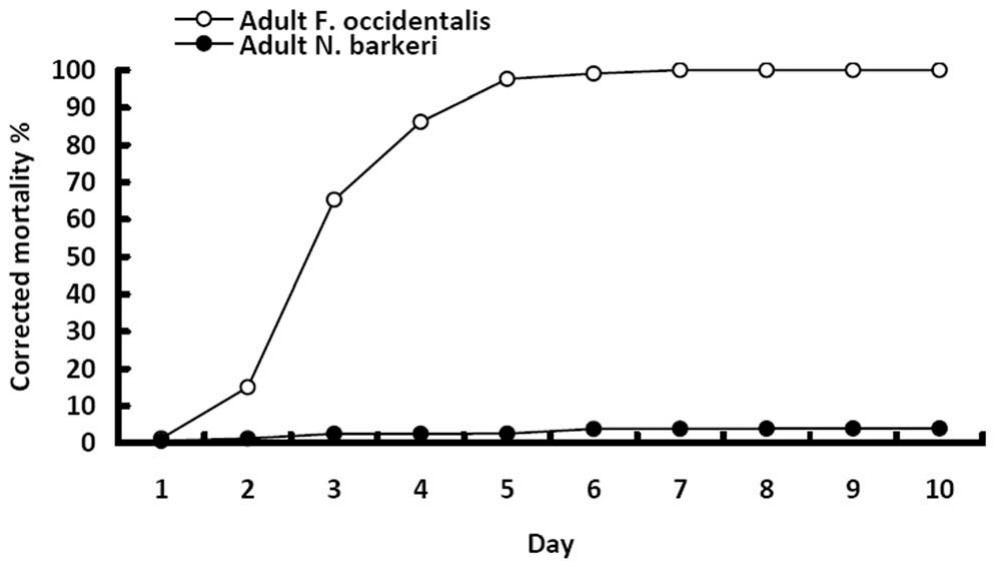
Corrected mortality of adult *F. occidentalis* and *N. barkeri* over 10 days following inoculation with 1×10^7^ conidia mL^1^ of *B. bassiana* strain SZ-26.

**Figure 3:**
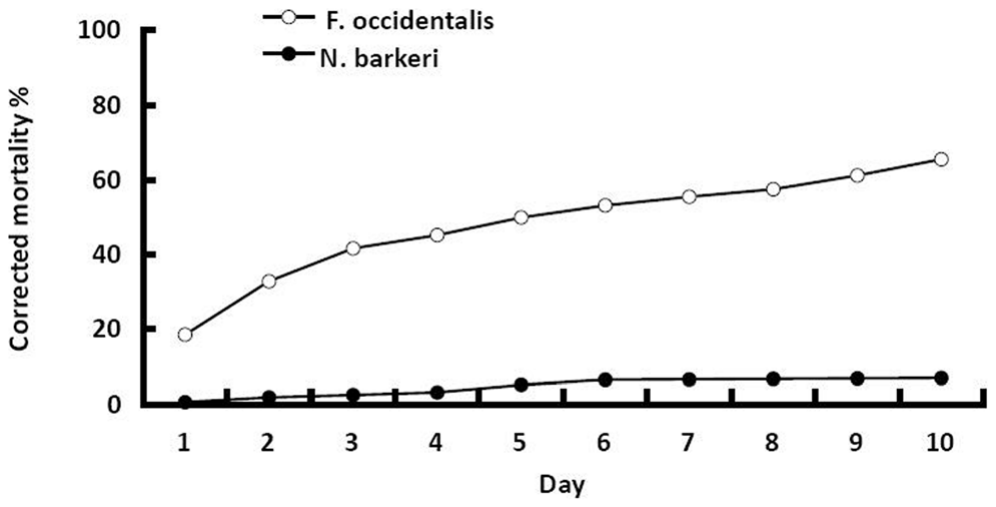
Corrected mortality of *F. occidentalis* and *N. barkeri* over 10 days following inoculation as first instars with 1×10^7^ conidia mL^1^ of *B. bassiana* strain SZ-26. After 10 days, surviving *F. occidentalis* had reached the pupal stage and surviving *N. barkeri* had reached the adult stage.

The corrected mortalities of *N. barkeri* were maintained below 4 and 8% at 10 days following inoculation of the adult and larvae, respectively. These mortalities for *N. barkeri* were significantly lower that those of *F. occidentalis*, whose corrected mortalities reached 100% and 66%, respectively, (Adult: t = 82.186, p<0.001; First instar: t = 57.531, p<0.001) (Fig. 2 and Fig. 3). No penetration of germ tube or formation of hyphal bodies was observed from dead predators as viewed under an optical microscope.

Effect of *B. bassiana* strain SZ-26 on the predator longevity and oviposition

When inoculated by *B. bassiana* strain SZ-26, preoviposition duration of predators was significantly longer as compared to the controls. There were no differences in other life table parameters, such as oviposition, postoviposition duration, female longevity and daily fecundity compared to the controls (Table 2).

**Table 2:** Length of reproductive durations, longevity (days ± SE) and fecundity (eggs ± SE) of *N. barkeri* when treated with *B. bassiana* strain SZ-26.

Length of reproductive durations, longevity and fecundity					
	Preoviposition	Oviposition	Postoviposition	Female Longevity	Daily fecundity
Untreated	2.48±0.12a	26.58±3.28a	7.12±0.47a	36.19±0.90a	1.90±0.05a
Treated	3.35±0.17b	25.00±3.03a	6.00±0.38a	34.32±0.56a	1.79±0.03a
df	45	49	49	41	49
t	−4.036	1.783	1.843	1.763	1.911
p	<0.001	0.081	0.071	0.085	0.062

Note: The same small letters in the same column represented no significant difference at 0.05 levels by T-test.

### Scanning electron microscopic observation

**Figure 4:**
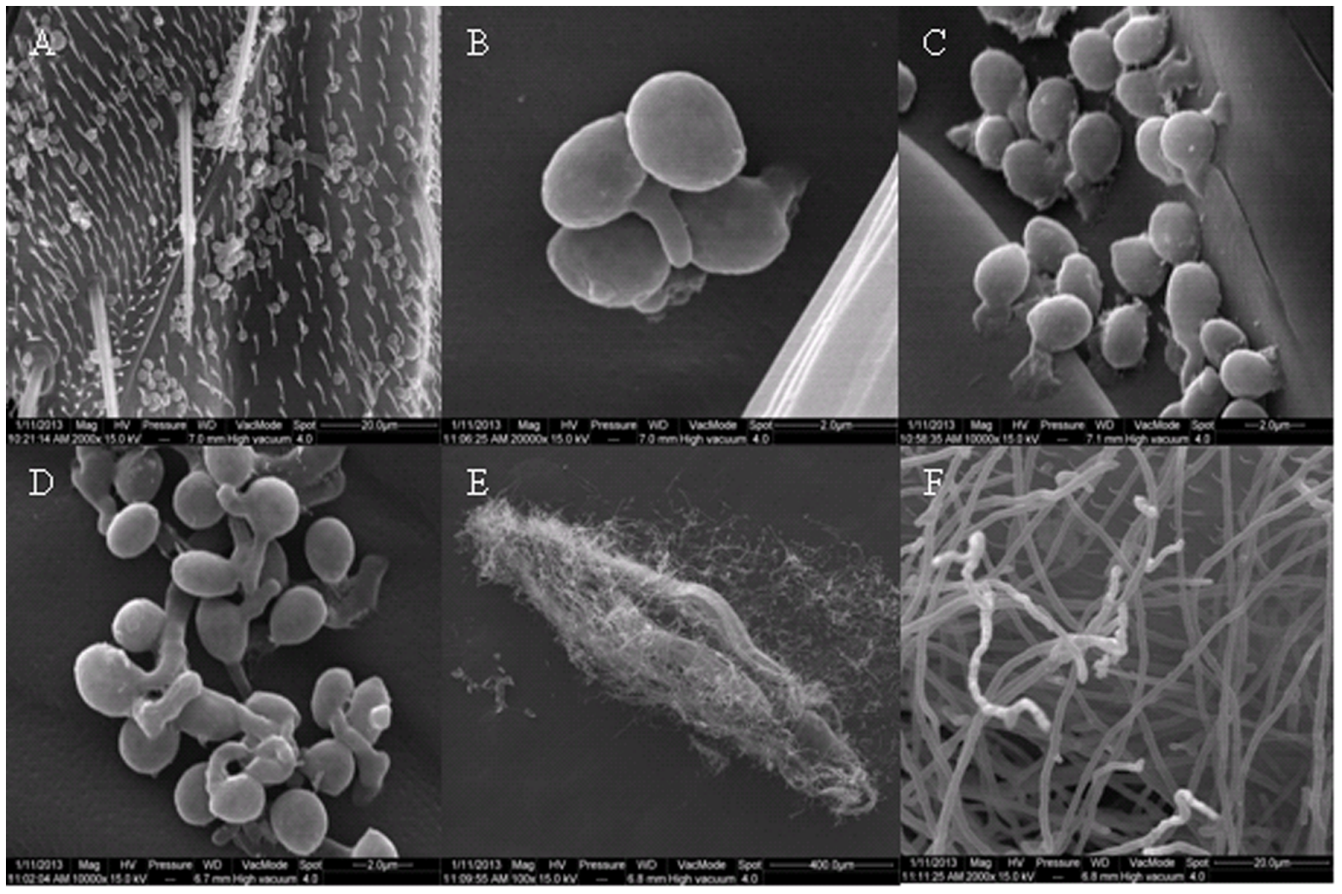
Germination and infection of *B. bassiana* strain SZ-26 conidia on the cuticle of *F. occidentalis*. (A) conidia adhering to the cuticle of *F. occidentalis*; (B) germ tube of conidia oriented toward cuticle; (C) germ tube penetratingthe cuticle; (D) fungal hyphae growing on the cuticle; (E) mycelium colonized the whole body; (F) conidia emerging from the dead adult.

When treated with 1×10^7^ conidia mL^−1^ of *B. bassiana* strain SZ-26, many conidia adhered to the cuticle of adult *F. occidentalis* after 2 h (Fig 4 A). Germ tubes of conidia oriented toward cuticle after 24 h (Fig 4 B). Germ tubes penetrated the cuticle after 36 h (Fig 4 C). Many conidia germinated and fungal hyphae were produced after 48 h (Fig 4 D). Mycelium colonized the whole body after 60 h (Fig 4 E). Conidia emerged from dead adults after 72 h (Fig 4 F).

**Figure 5:**
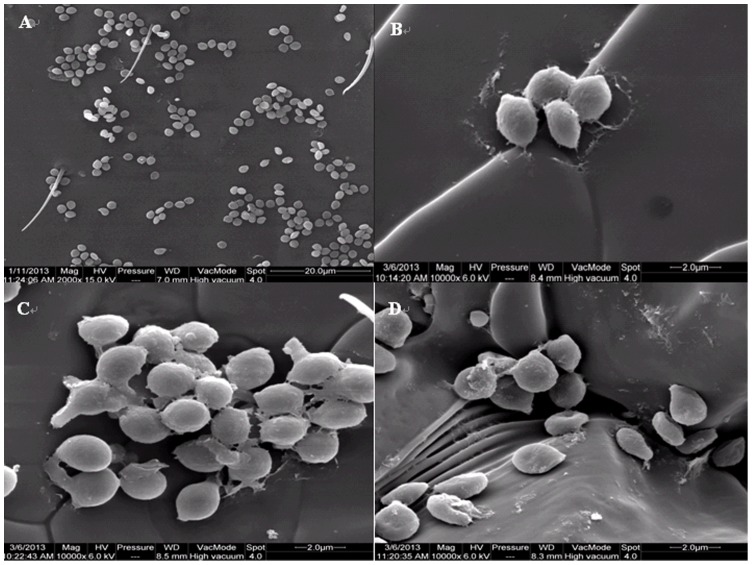
Inoculation and attachment of *B. bassiana* strain SZ-26 conidia on the cuticle of *N. barkeri*. (A) conidia adhering to the cuticle of *N. barkeri*; (B) secretion on the interface of conidia and cuticle; (C) germ tube of conidia oriented toward cuticle; (D) shriveled condia

When *N. barkeri* were treated with 1×10^7^ conidia mL^−1^ of *B. bassiana* strain SZ-26, the conidia could adhere to the cuticle of adults after 2 h (Fig 5 A). Secretions on the interface of conidia emerged after 12 h (Fig 5 B), Conidia germinated after 24 h, but were not observed to penetrate the cuticle within 36 h (Fig 5C). Conidia were shed gradually from the body, leaving the secretions on the surface of the cuticle. Several condia were observed to have shriveled after 48 h (Fig 5 D). Few conidia were detected on the body after 48 h.

## Discussion

Risk evaluation and compatibility research on pathogens and predators have always draw scientist's attention. Furtado et al. [30] reported that a strain of the fungal pathogen, *Neozygites acaricida* was pathogenic to a phytoseiid mite, *Euseius citrifolius*, while other studies have shown other fungal pathogens displayed no pathogenicity to predatory mites [9], [18], [23]. Our study used a novel strain of *B. bassiana* strain SZ-26 that is highly virulent to *F. occidentalis*, but proved not to be detrimental to both adult and larval *N. barkeri*. Although first instar thrips are considered the most susceptible life stage to entomopathogenic fungi compared to the other life stages [31], our results showed that adult thrips were more susceptible to *B. bassiana* strain SZ-26. This differential mortality may be because fungal conidia are shed with the exuvium following ecdysis decreasing pathogenicity to immature stages. These results are supported by the studies of Vestergaard et al. [32] and Maniania et al. [33] who also demonstrated that the mortality of entomopathogenic fungus on adult *F. occidentalis* were displayed much higher than for larvae.

There has been increasing interest in evaluating the sub-lethal effects of pathogens on predators. Shaw et al. [34] reported that the fecundity of the predatory mites *Euseius hibisci*, *Amblyseius limonicus* and *Typhlodromus occidentalis* are not affected by feeding on virus-infected citrus red mites, *Panonychus citri*. *Neozygites floridana* does not affect the oviposition of *Phytoseiulus longipes* when fed with *N. floridana* infected *Tetranychus evansi* and *Tetranychus urticae*. [23]. While the longevity and fecundity of predatory mites *Phytoseiulus persimilis* were displayed lower when fed on *B. bassiana* treated spider mite, *Tetranychus urticae* for 24–72 h [35]. We observed *N. barkeri* could not only feed on *B. bassiana*-infected larval *F. occidentalis*, but also feed *B. bassiana* strain SZ-26 conidial suspension directly (unpublished). In this study, the sub-lethal effects on *N. barkeri* when directly exposed to *B. bassiana* strain SZ-26 conidial suspension were evaluated. we observed that *N. barkeri* groomed conidia from their bodies so that few conidia remained on *N. barkeri* 48 h after treatment with *B.* bassiana strain SZ-26 (unpublished). One function of grooming in arthropods is the removal of foreign bodies such as fungal or mite parasites [36]. Wekesa et al [23] reported that the predatory mite, *Phytoseiulus longipes* was efficient in removing most capilliconidia of the fungal pathogen *N. floridana* through self-grooming behavior.

In order to avoid being influenced by other factors, we supplied untreated larval *F. occidentalis* to predators as food, because *B. bassiana* infection could make the larval thrips deficient in certain essential nutrients [37] that may reduce fecundity of female predator, or create a buildup of fungal toxins or metabolites that may shorten adult predator longevity [38]. Whether feeding infected larvae of *F. occidentalis* to *N. barkeri* will affect life table parameters of the predator still needs to be demonstrated. From our recent results, this exposure did lengthen the preoviposition period of adult females. However, this likely reflects time spent by all treated mites grooming off conidia and not a physiological effect on females. Overall, both direct bioassay and sub-lethal effects on *N. barkeri* indicated that *B. bassiana* strain SZ-26 poses a negligible risk to *N. barkeri*.

From our SEM observations, *B. bassiana* strain SZ-26 conidia penetrated *F. occidentalis* cuticle soon after germination. The results agree with those of Vestergaard et al. [32] and Wang et al. [39] in their studies with most fungus germlings producing appressoria within 24–48 h post-inoculation on *F. occidentalis*. In contrast, despite being able to attach to *N. bakeri*, it was displayed that no pathogenicity of *B. bassiana* strain SZ-26 to *N. bakeri*. No penetration of germ tube or formation of hyphal bodies was observed on dead *N. bakeri* further supporting the SEM results. The pathogenicity of entomopathogenic fungi is the result of mechanism pressure and proteinases which are associated with cuticle degradation [40]–[44]. This raises questions regarding the capacity of *N. bakeri* to avoid infection by fungi. Although many studies indicate that entomopathogenic fungi are highly pathogenic against targeted insect pests while showing no detrimental effects on predators in laboratory bioassays and field investigations [9], [27], it is unclear how entomopathogenic fungi identify and infect hosts species. In our study, most conidia was removed by self-grooming off the *N. bakeri* body within 48 h, reducing the infection possibility. Moreover, although conidia could germinate, they were not observed to penetrate the *N. bakeri* cuticle, we speculate that the different cuticle structures or proteinase targets between *F. occidentalis* and *N. bakeri* influence the fungi pathogenicity. The proteinaceous outer integument of predatory mites probably forms an effective barrier against *B. bassiana* strain SZ-26. In addition, few shriveled conidia were detected on the cuticle of *N. barkeri* after 48 h, possibly because the germinated conidia which were remaining on *N. bakeri* could not be glued on the susceptible host, the few shriveled conidia probably lost their viability. These observations and speculations may aid in explaining why *N. bakeri* in not infected by *B. bassiana* strain SZ-26. The results also enhance our understanding of the interactions between pathogen and predators. To better understand the interactions, the defense mechanisms of predators need to be further explored.

## Materials and Methods

### Subheading Ethics Statement

No specific permissions were required for these locations/activities.

None of the species used in this study are endangered or protected.

### Beauveria bassiana

The origin and source of the twenty-eight fungal isolates are shown in Table 1. All isolates were maintained and conidia were produced on Sabouraud Dextrose Agar (SDA) at 26±1°C under continuous darkness. Conidial concentrations were determined with a hemocytometer and adjusted with sterile water containing Tween-80 at 0.05% (v/v). The viability of the conidia was confirmed on SDA medium [25] and was >90% for all strains.

### Mite colony

*N. barkeri* and the prey *Tyrophagus putrescentiae* were obtained from colonies maintained in the laboratory of insect natural enemies, Institute of Plant Protection, Chinese Academy of Agricultural Sciences. *N. barkeri* are reared in sterilized wheat bran-*T. putrescentiae* mixture and fed on *T. putrescentiae* in plastic boxes (15 cm×15 cm×10 cm) with lips and a circular moist sponge (10 cm diameter) at the edge of boxes for preventing escape. A hole (12 cm diameter) was cut in the lid and covered with fine mesh to allow for ventilation. Culture boxes were kept at 25±1°C, 60–70% RH and L16:D8 photoperiod in a climate controlled chamber. Cotton silk was placed on the surface of the leaves for oviposition, eggs were collected and transferred to a new plastic box using a fine paintbrush after 6 hours and allowing the emergent larvae to develop in synchrony. The newly emerged larvae and adults were obtained for experimental use.

### Western flower thrips colony

A colony of western flower thrips, *F. occidentalis* was maintained as described by Liang et al [26]. Briefly, thrips colonies were continuously reared on sterilized kidney beans (*Phaseolus vulgaris* L.) in 0.5 L tube-shaped glass jars with snap-on lids. A hole (10 cm diameter) was cut in the lid and covered with fine mesh to allow for ventilation. Rearing jars were kept at 26±2°C, 60–70% RH and L13:D11 photoperiod in a climate controlled chamber. Thrips at similar stages of development were obtained by incubating adults on fresh, healthy plants for oviposition, removing the thrips after 3 days and allowing the different stage of thrips to develop in synchrony. The first instars and adults were obtained for experimental use.

### Screening of 28 new fungal isolates

The effect of the fungal isolates on adult *F. occidentalis* survival was evaluated by treating thrips with concentrations of 1×10^7^ mL^−1^ conidia, which is the concentration commonly used for spray application for control of western flower thrips in greenhouses in China [27]. A control consisted of sterile water containing Tween-80 at 0.05% (v/v). Individual newly eclosed *F. occidentalis* adults were collected from the laboratory rearing colony and dipped for 5 s in the conidial suspension. Adults were allowed to dry on filter paper and transferred to Petri dishes (diameter 7 cm) lined with bean leaves and covered with plastic film which were pricked for ventilation. The Petri dishes were stored in a climate cntrolled chamber (26±2°C, RH 60–70% and 13 L: 11D photoperiod). The effects against the *F. occidentalis* adults were scored at day 5 after treatment. The presence of fungal mycelia was used as an indication of mycosis. Each replicate consisted of 20 adults; treatments were randomized and the experiment was replicated 3 times using different insect lots over time.

### Efficacy against *F. occidentalis* and *N. barkeri* with the SZ-26 strain

Based on the screening of the 28 new strains as reported above, strain SZ-26 was re-evaluated against *F. occidentalis* and *N. barkeri* using the same conditions listed above. The first instar larval and newly eclosed adult stages of *F. occidentalis* were inoculated by immersion for 5 s in 2 ml conidia suspension of *B. bassiana* strain SZ-26 and using a fine paintbrush carefully transferred to petri dish (3.5 cm diameter) lined with freshly excised bean leaf, which was placed on the surface of the water—saturated filter paper, the root vein of leaf was wrapped by moist cotton wool to slow leaf desiccation. The dish was then sealed with polyvinyl chloride (PVC) cling film and incubated at 25±1°C, 60–70% RH and L16:D8 photoperiod in a climate controlled chamber. The status of individuals was determined 10 days after treatment. Mortality was recorded daily. Each stage of thrips consisted of 8 replicates with 20 insects per replicate. The presence of fungal mycelia was used as an indication of mycosis. Controls consisted of thrips treated with 0.05% Tween-80 in sterile H_2_O. Bioassays for adult and larval *N. barkeri* were repeated as described above. Ample *T. putrescentiae* immatures were needed to supply as food, the dead *N. barkeri* were picked and placed on SDA at 26±1°C under continuous darkness, then examined under optical microscope for the presence of *B. bassiana* strain SZ-26 conidia or hyphal bodies. Each replicate consisted of 20 adult *N. barkeri*, treatments were randomized and the experiment was replicated 8 times using different insect lots over time.

### Effect of SZ-26 strain on the predator longevity and oviposition

The experimental units were designed with two pieces of uniform organic glass (6 cm×5 cm×4 mm), the water—saturated filter paper was placed on one piece, the freshly excised leaf of kidney beans was upside down on the surface of the filter paper, a hole (2.5 cm diameter) was punched in another piece and pressed on the leaf. A chamber was formed between two pieces of organic glass which served as the experimental platform. The newly molted female adults were inoculated by immersion for 5 s in 2 ml conidial suspension of *B. bassiana* strain SZ-26 and placed individually in each chamber and about 20 first-instar larval *F. occidentalis* were supplied as food. A male was added to each chamber for 1 d to allow mating and then the male was removed. The successfully mated females started to lay eggs, the daily fecundity of each was recorded until the females died, predators were transferred into new chambers and supplied daily with first instars as food. The excised leaves were changed every 4–5 days and the predators were transferred into new chambers. The oviposition period and female longevity were also estimated. Controls were set up only with untreated females. For treatment and control, a total of 30 synchronized female predators were tested.

### Scanning electron microscope observations (SEM)

For SEM observation, the predatory mites and thrips were collected and inoculated by immersion for 5 s in 2 ml conidial suspension of the SZ-26 strain, then transferred into the chamber (10/species in each chamber). Ample *T. putrescentiae* immatures were supplied to predators as food. After 1, 2, 12, 24, 36, 48, 60 and 72 h, the SZ-26 -treated samples were fixed in 70% ethyl alcohol for 24 h, than dehydrated in a ascending series of ethyl alcohol (75, 80, 90, 95 and 100%, 6 min each), left to air dry for a few seconds and mounted on SEM stubs with double-sided carbon tape. Dried samples were sputtered with gold and observed with the SEM under Quanta 200 FEG at high-vacuum mode.

### Statistical analysis

All statistical analyses were carried out using SPSS software [28]. Data of mortality were corrected for control mortality [29] and were normalised using arcsine transformation. Differences of mortality between two species were evaluated using a T-test procedure at a = 0.05 to determine significance. Differences of longevity and oviposition between treatment and control were also compared by T-test after log transformation of the data. Data will be available from the corresponding author upon request.
